# Enhanced performance of lead sulfide quantum dot-sensitized solar cells by controlling the thickness of metal halide perovskite shells

**DOI:** 10.1016/j.heliyon.2023.e20276

**Published:** 2023-09-19

**Authors:** Gabseok Seo, Shinhyun Kim, Hyunseok Choi, Min-cheol Kim

**Affiliations:** a Frontier Energy Solution Corporation, Seoul National University, Seoul 08826, Republic of Korea; b School of Mechanical Engineering, Pusan National University, Busan 46241, South Korea

**Keywords:** Lead sulfide quantum dot, Quantum dot solar cell, Metal halide perovskite, Core/shell, Carrier dynamics

## Abstract

The metal halide perovskite CH_3_NH_3_PbI_3_ (MAP) can be applied as the shell layer of lead sulfide quantum dots (PbS QDs) for improving solar power conversion efficiency. However, basic physics for this PbS core/MAP shell QD system is still unclear and needs to be clarified to further improve efficiency. Therefore, in this study, we investigate how MAP shell thickness affects device performance and dynamics of charge carriers for PbS QD-sensitized solar cells. Covering the PbS QDs with the MAP shell layers of an appropriate thickness around 0.34 nm greatly suppresses charge carrier recombination at surface defects along with improved carrier transport to neighboring oxide and polymer layers. Therefore, this MAP shell thickness provides the highest open-circuit voltage, short-circuit current density, and fill factor for solar cells. Overall power conversion efficiencies of these solar cells reached about 4.1%, which is about six-fold higher than that for solar cells without MAP (about 0.7%). Additionally, use of the MAP shell layers can prevent oxidation of PbS QDs and, therefore, makes a degradation of solar cell performance slower in air.

## Graphical abstract

**Figure:**
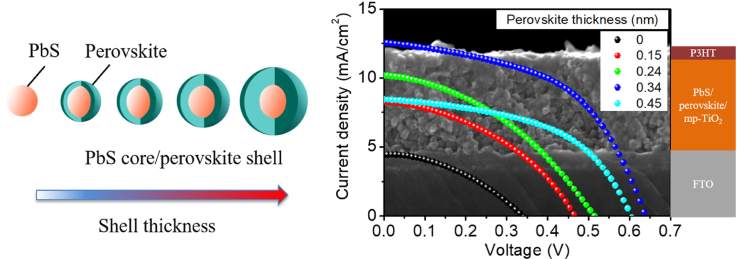

## 1 Introduction

Since the discovery of dye-sensitized solar cells (DSSC) by Grätzel et al. [[1], [2], [3]], their device performance has rapidly improved to a level for practical applications. Quantum dots (QDs) have also been used as the sensitizer for solar cells because of their unique properties, such as bandgap tunability, high extinction coefficient, and low-cost solution processing [[4], [5], [6], [7], [8]]. However, QD-sensitized solar cells still have power conversion efficiency (PCE) lower than a theoretical maximum value which is originated from defects acting as carrier traps and recombination centers at QD surfaces [[9], [10], [11], [12], [13], [14]]. As for solar cells sensitized by lead sulfide (PbS) QDs used in this study, surface defects are also a factor that strongly limits solar cell performance [15]. The presence of surface defects causes instability of QDs and solar cells to be degraded [16,17]. It is widely known that coating QDs with thin shell layers is very effective in passivating surface defects, which makes it possible to prevent carrier recombination and enhance charge carrier extraction and stability for QD-sensitized solar cells [[18], [19], [20]].

We recently demonstrated that PbS QDs coated with shell layers of the metal halide perovskite CH_3_NH_3_PbI_3_ (MAP) are directly formed inside a mesoporous TiO_2_ (mp-TiO_2_) layer and that PbS QD-sensitized solar cells with MAP shell layers have improved solar cell performance when compared with those without MAP shell layers [17]. To sufficiently passivate surface defects and reduce carrier recombination for PbS QDs, an increase of MAP shell thickness is necessary. However, too large MAP shell thickness is expected to impede carrier extraction from devices because of increased electrical resistance. Therefore, it is very important to optimize MAP shell thickness. However, the optimization of MAP shell thickness for PbS QD-sensitized solar cells has not been performed yet to date. In this study, we investigated the effect of MAP shell thickness on performance of PbS QD-sensitized solar cells. Additionally, we carried out time-resolved photoluminescence spectroscopy (TRPL), transient photovoltage (TPV) measurement, and X-ray photoemission spectroscopy (XPS) on PbS QDs with different MAP thicknesses for a better understanding of carrier recombination and extraction behaviors in solar cells. We found that use of a certain MAP shell thickness around 0.34 nm leads to a greatly improved power conversion efficiency of 4.1% because of reduced carrier recombination along with enhanced carrier extraction. The MAP shell layers provided another merit that stability of solar cells is improved in air by preventing the oxidation of PbS. These results would contribute toward fabrication of PbS QD-sensitized solar cells with high efficiency and air stability.

## 2 Experimental

### 2.1 Preparation of TiO_2_ films

On top of glass substrates coated with a fluorine-doped tin oxide (FTO) layer (Pilkington, TEC8), a compact TiO_2_ (c-TiO_2_) layer with a thickness of *ca*. 50 nm was prepared by a spray pyrolysis deposition method using 20 mM solution of titanium diisopropoxide bis(acetylacetonate) (Sigma Aldrich) in ethanol at 450 °C. The c-TiO_2_ layer plays a role in preventing formation of shunting paths between electrodes. Paste containing anatase-phase TiO_2_ nanoparticles with an average diameter of ca. 50 nm was then screen-printed on the c-TiO_2_ layer to prepare an mp-TiO_2_ layer with a thickness of ca. 1 μm [21,22] and annealed at 500 °C for 1 h in an air. The mp-TiO_2_ layer was subsequently submerged in a 20 mM TiCl_4_ solution at ambient temperature for 12 h, rinsed with distilled water, and then sintered at 450 °C for 15 min to enhance the contact between the TiO_2_ particles.

### 2.2 Synthesis of MAI

MAI was synthesized by reacting 30 mL of methylamine (40% in methanol, TCI) and 32.3 mL of hydroiodic acid (57 wt% in water, Aldrich) in 250 mL round-bottom flask at 0 °C for 3 h with stirring. The precipitate was obtained by removing the solvents using a rotary evaporator at 50 °C. The obtained yellowish raw product (MAI) was washed with diethyl ether three times and finally purified by recrystallization from a mixed solvent of diethyl ether and ethanol. After filtration, the white MAI powder was dried at 60 °C in a vacuum oven for 24 h.

### 2.3 Film and device fabrication

Film fabrication was performed *via* spin-assisted successive ionic-layer-adsorption reaction (S-SILAR) process. EDT molecules behave to terminate PbS QD surfaces for stabilization and prevent overgrowth of PbS QDs [11]. Next, MAP shell layers were formed on PbS QD surfaces by a two-step method, which includes spin-coating a PbI_2_ solution, followed by spin-coating a methylammonium iodide (MAI) solution [23]. After baking the films at 70 °C, we observed a rapid change of film color to dark brown, indicating the formation of the MAP structure (Figure S1) as reported in literature [24]. To deposit PbS QDs on the mp-TiO_2_ layer by the S-SILAR method, we prepared 100 mL of a 5 mM PbI_2_ (Sigma–Aldrich) solution in *N,N-*dimethylformamide (DMF, Sigma-Aldrich), 100 mL of a 5 mM Na_2_S (Aldrich) solution in methanol (Burdick & Jackson)/deionized water (95:5 in volume), and 100 mL of an EDT/acetonitrile (1:99 in volume) solution. We successively spin-coated the PbI_2_, Na_2_S, EDT solutions on the mp-TiO_2_ layer. The solution amount used for each spin-coating was 100 μL and each spin-coating speed was 1500 rpm for 20 s. The above spin-coating procedure was repeated 20 times to incorporate PbS QDs into the mp-TiO_2_ layer. To form MAP shell layers on the PbS QDs, the mp-TiO_2_ layer was first infiltrated with PbI_2_ by spin-coating 200 μL of a PbI_2_ solution in DMF at 4000 rpm for 30 s and drying at 70 °C for 30 min under argon atmosphere. Then, 100 μL of the solution of MAI (0.035 M; 2-propanol) was dropped over the layer and dried at 70 °C for 10 min [25]. On top of the core/shell QD layer, a hole transport layer was deposited by spin-coating poly-3-hexylthiophene (P3HT, Rieke metals, >98% region regularity) solution of a 15 mg mL^−1^ concentration in 1,2 dichlorobenzene at 2500 rpm for 60 s. The solution of PEDOT:PSS [poly(3,4-ethylenedioxythiophene) polystyrene sulfonate] (Clevios, Al4083) was diluted with double the amount of methanol and spin-coated on the P3HT layer at 2000 rpm for 30 s. To finalize the solar cells, an 80-nm-thick gold electrode layer was thermally evaporated on the PEDOT:PSS layer under a pressure of 5 × 10^−6^ Torr. To improve electrical connections, we attached leads to both the FTO and Au electrodes using an ultrasonic soldering iron (USS-9200, MBR Electronics). The measured active area for the device was 0.16 cm^2^.

### 2.4 Film and device characterization

The current density voltage (*J–V*) characteristics were recorded utilizing a 3A Class AAA solar simulator (Newport, 64023A) paired with a 450 W xenon lamp (Newport, 6279NS) and interfaced with a potentiostat (CH Instruments, CHI 600D). For all devices, the *J*–*V* profiles were obtained by limiting the active region using a metal mask that covers 0.096 cm^2^. The illumination intensity was calibrated with a standard Si-reference cell (Oriel, VLSI standards), where 1 sun corresponds to 1000 W m^−2^. EQE measurements in the wavelength range of 300–800 nm were conducted with a fully automated system, which included a 300 W xenon lamp (Newport, 66902) with monochromator (Newport, Cornerstone 260), and a multimeter (Keithley, 2002). The EQE values in a longer-wavelength region from 500 to 1700 nm were measured using the same setup mention earlier, except a 1000 W xenon lamp (Newport, 69935) was used. For EQE measurements, light intensity calibration at each wavelength was conducted using either a Si photodetector or an InGaAs photodetector. TRPLS decay curves were derived using an optical parametric oscillator laser (Spectra-Physics, basiScan), energized by Nd-YAG (Spectra-Physics, INDI-40-10). The excitation pulse had a duration of 7 ns, with its energy modulated between 1.0 and 0.1 mJ per pulse *via* neutral density filters. Decay curves were captured at the emission peak for each specimen utilizing a NIR photomultiplier tube (Hamamatsu, H10330-75) coupled with a monochromator (Princeton Instruments, SP2300). The photomultiplier output was logged using a 500 MHz digital oscilloscope (Agilent, DSO X-3054A). A cut-off filter, situated ahead of the photomultiplier, eliminated any scattered excitation light. TPV decay assessments were carried out employing a 10 Hz ns laser (EKSPLA, NT342A-10) for slight perturbation illumination and a 150 W Xe lamp (Zolix) as a bias light source. The instrument was directly interfaced with a 500 MHz digital oscilloscope (Agilent DSO X-3054A), with its input impedance configured to 1 MΩ in an open-circuit condition. Neutral density filters modulated the bias light intensity for assorted *V*_*OC*_, and a considerably attenuated laser pulse at 550 nm generated a voltage transient, not surpassing 20 mV. XPS evaluations of chemical states were conducted using the Quantum 2000 system, utilizing an Al Kα X-ray source (1486.6 eV) and a hemispherical electron analyzer. The derived XPS data offered mean chemical details over a spatial expanse of 100 μm in diameter and a depth between 5 and 10 nm.

## 3 Results and discussion

**Fig. 1:**
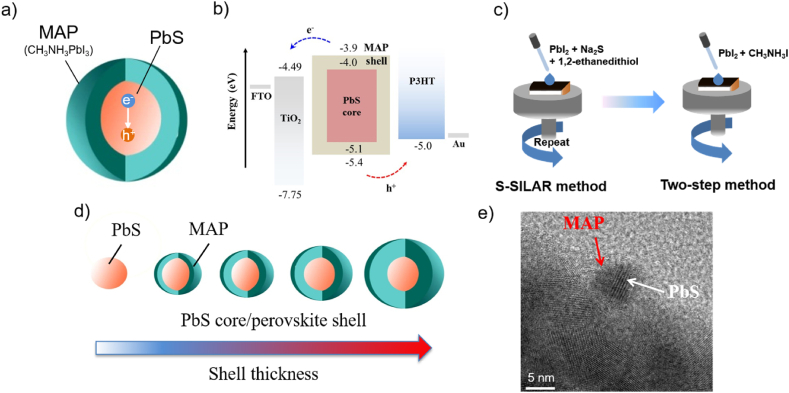
(a) Schematic illustration of a PbS core/MAP shell QD. (b) Energy-level diagram of QD-sensitized solar cells fabricated in this study. Illustrations showing (c) S-SILAR and two-step methods for the fabrication of PbS core/MAP shell QDs. (d) MAP shell thickness can be controlled by PbI_2_ solution concentrations used for the two-step method. (e) TEM image of PbS core/MAP shell QDs with a diameter of 3.0–5.5 nm.

The architecture of PbS QD-sensitized solar cells fabricated in this study is glass substrate/fluorine-doped tin-oxide (FTO)/compact TiO_2_ (c-TiO_2_)/sensitizer-incorporated mesoporous TiO_2_ (mp-TiO_2_)/poly(3-hexylthiophene-2,5-diyl) (P3HT)/poly(3,4-ethylenedioxythiophene) polystyrene sulfonate (PEDOT:PSS)/Au, where the sensitizer is PbS QDs covered with MAP shell layers as shown in Fig. 1a. The energy-level diagram of these devices is shown in Fig. 1b. Electron-hole pairs generated in the PbS QDs under solar illumination are separated into free electrons and holes, which are transported into mp-TiO_2_ and P3HT, respectively. These electron and hole transport through the MAP shell layers seem efficient in terms of the similar hole and electron transport levels for PbS and MAP. The MAP shell layers play an important role in reducing carrier recombination by passivating surface defects of PbS QDs, reducing electrons coming back from mp-TiO_2_, and preventing a direct contact between mp-TiO_2_ and P3HT, the source of shunting paths.

The PbS core/MAP shell QDs used for the above devices were prepared using methods reported previously and illustrated in Fig. 1c [17,23]. PbS QDs were directly formed inside an mp-TiO_2_ layer by repeated spin-coating of three solutions each containing PbI_2_, Na_2_S, or 2-ethanedithiol (EDT) as described in experimental section in detail [17]. To control MAP shell thickness, different concentrations of PbI_2_ solutions were used for the two-step process while the MAI solution concentration was fixed. The PbI_2_ solution concentrations ranged from 10, 30, 50, and 100 mM. As illustrated in Fig. 1d, larger MAP shell thicknesses can be obtained when the higher PbI_2_ solution concentrations are used, which will be discussed later.

PbS core/MAP shell QDs were removed from mp-TiO_2_ films by scratching using a knife and suspended in anhydrous ethanol (Sigma-Aldrich) by ultrasonication. The QD suspension was dropped onto a grid and dried for transmission electron microscopy (TEM). Fig. 1e shows a TEM image of PbS core/MAP shell QDs, which were fabricated using the 50 mM PbI_2_ solution for the two-step process. The white and red arrows in this TEM image indicate the PbS core and the MAP shell layer, respectively. The average PbS core diameter was about 3.7 nm. The crystalline structures each for the PbS core and the MAP shell were already discussed in our previous paper [17].

**Fig. 2:**
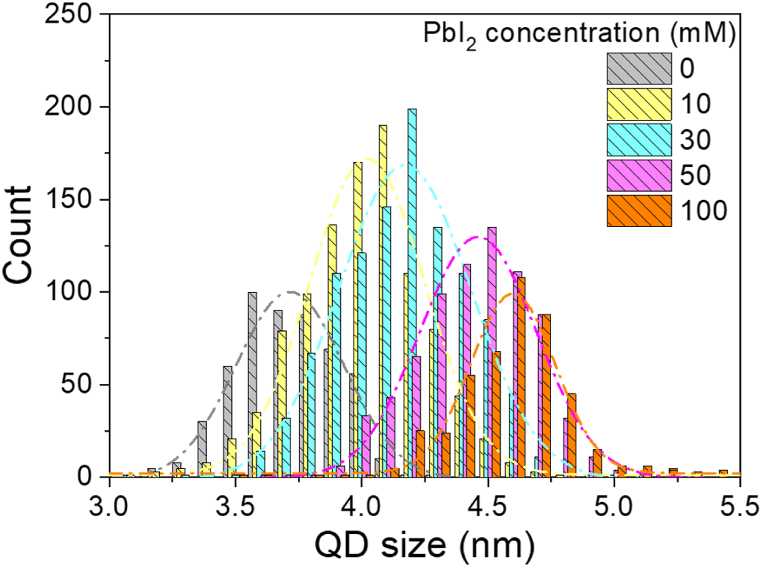
Distributions of QD sizes calculated from TEM images.

We performed the same TEM measurements on other PbS core/MAP shell QDs fabricated using the different PbI_2_ solution concentrations, and analyzed their size distributions using open-source software ImageJ [26,27]. The obtained QD size distributions are shown in Fig. 2. The average QD diameters were 4.0 nm for the 10 mM solution, 4.18 nm for the 30 mM solution, 4.38 nm for the 50 mM solution, and 4.60 nm for the 100 mM solution, respectively. By taking the PbS core diameter into account, the average MAP shell thicknesses could be estimated to be 0.15 nm for the 10 mM solution, 0.24 nm for the 30 mM solution, 0.34 nm for the 50 mM solution, and 0.45 nm for the 100 mM solution, respectively. It is clear that use of the PbI_2_ solutions with the higher concentrations results in an increase of MAP shell thickness. We hereafter used these average MAP thicknesses for the discussion.

**Fig. 3:**
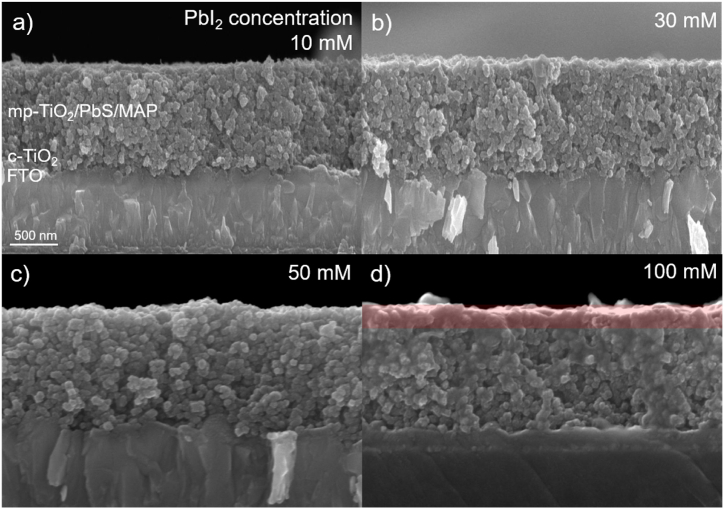
Cross-sectional SEM images of PbS core/MAP shell QD samples fabricated using (a) 10, (b) 30, (c) 50, and (d) 100 mM PbI_2_ solutions. In the red region of (d), pores are filled with excess MAP.

Fig. 3 shows cross-sectional scanning electron microscope (SEM) images of mp-TiO_2_ films with PbS core/MAP shell QDs. There were tiny pores in the whole regions of the mp-TiO_2_ films when the 10 (Figs. 3a), 30 (Fig. 3b), and 50 mM (Fig. 3c) PbI_2_ solutions were used to form the MAP shell layers. These pores could be filled with P3HT when a P3HT layer is spin-coated on top of the mp-TiO_2_ films. As for the mp-TiO_2_ films fabricated using the 100 mM PbI_2_ solution (Fig. 3d), the pores still exist near the c-TiO_2_ surface, but were not clearly observed near the mp-TiO_2_ film surface. This could be because of filling the pores with excess MAP. In this case, P3HT molecules cannot reach the lower part of the mp-TiO_2_ films to fill the pores. Therefore, the holes generated under illumination cannot move properly to a hole transport layer of P3HT, probably making solar cell performance lower [[28], [29], [30]].

**Fig. 4:**
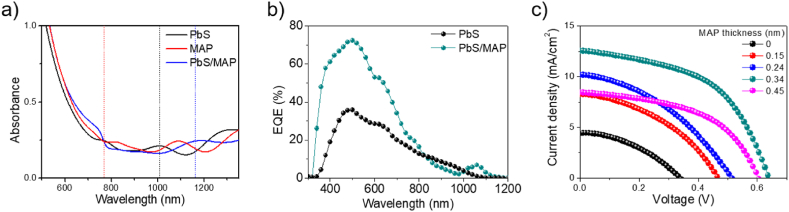
(a) Vis-NIR absorption spectra of PbS QDs, MAP, and PbS core/MAP shell QDs film on a FTO substrate prepared by S-SILAR method. (b) EQE curves of PbS QD-sensitized solar cells with and without MAP shell layers of 0.34 nm. (c) *J-V* curves of PbS QD-sensitized solar cells with various MAP shell thicknesses.

We prepared three kinds of samples for UV–Vis absorption spectroscopy. The first sample was an mp-TiO_2_ film with PbS QDs fabricated using the S-SILAR method only (no MAP included). The second sample was an mp-TiO_2_ film with MAP fabricated using the two-step method only (no PbS QD included). The third sample was an mp-TiO_2_ film with PbS core/MAP shell QDs fabricated using both the S-SILAR and two-step methods. To fabricate the second and third samples, the 50 mM PbI_2_ solution was used. Fig. 4a shows the absorption spectra of these three samples. Absorption wavelengths of PbS QDs depend on QD diameters and are typically in the near-infrared region between 700 and 1400 nm [31]. Our PbS QDs without the MAP shell layers had an absorption peak at about 1020 nm. The PbS QD diameter estimated from this absorption wavelength using a method reported previously was 3.6–4.0 nm, which agrees with that estimated from the TEM images [10]. It is worth mentioning here that covering the PbS QDs with the MAP shell layers causes the absorption peak to redshift from 1020 to 1170 nm. This may mean an increase of the MAP shell thickness. For the MAP sample without the PbS QDs, the absorption originating the MAP structure was located at about 780 nm. The similar peak was observed in the PbS core/MAP shell QD sample. In addition to the absorption peaks discussed earlier, these absorption spectra contained other peaks, which could be caused by optical interference effects.

External quantum efficiency (EQE) curves of PbS QD-sensitized solar cells in the absence and presence of MAP shell layers are presented in Fig. 4b. The shapes of the two EQE curves were very similar, indicating that MAP does not contribute to solar power conversion in our QD systems although metal halide perovskites can be widely used as the light absorber of solar cells [23,[31], [32], [33]]. Introduction of MAP shell layers into PbS QDs led to an increase of EQE by about twice, the reason of which is discussed later.

Current density-voltage (*J-V*) characteristics of PbS QD-sensitized solar cells with the different MAP shell thicknesses were measured under AM 1.5G solar illumination at 100 mW cm^−2^ and are shown in Fig. 4c. To increase the statistical significance of our efficiency data, 50 samples were fabricated for each and measured; the photovoltaic parameters, short circuit current density (*J*_*SC*_), open circuit voltage (*V*_OC_), fill factor (FF), and PCE, obtained from the *J*-*V* characteristics are summarized in Table 1 and plotted as a function of MAP thickness in Figure S2. These parameters strongly depended upon the MAP thicknesses (the PbI_2_ solution concentrations) and became maximum at a MAP thickness of 0.34 nm; the value was about 4.1%. This value was about six-fold higher than that for solar cells without MAP (about 0.7%)

**Table 1:** Summary of device performance of PbS QD-sensitized solar cells with different MAP shell thicknesses.

MAP thickness (nm)	*J*_*SC*_ (mA/cm^2^)	*V*_*OC*_ (mV)	FF	PCE (%)
0 (ref. 17)	6.0	343	35.2	0.7
0.15	8.3	460	40.8	1.6
0.24	10.2	510	38.9	2.0
0.34	12.5	630	51.5	4.1
0.45	8.5	600	50.5	2.6

**Fig. 5:**
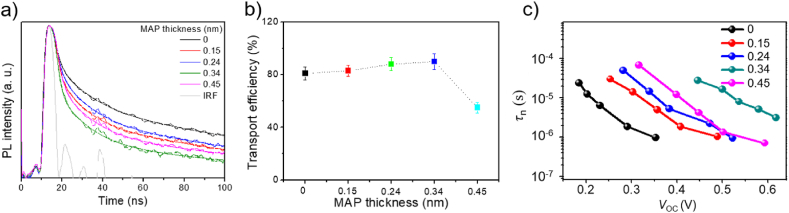
(a) TRPL decay curves of PbS QDs with different MAP shell thicknesses. (b) Plot of electron transport efficiency as a function of MAP shell thickness. (c) Recombination lifetime (*τ*_n_)-*V*_*OC*_ plots for solar cells.

To understand a reason for the improved solar cell performance, we carried out TRPL on PbS QD samples because TRPL results can provide us important information on dynamics of photo-excited carriers [34]. For TRPL measurements, we prepared mp-TiO_2_ films with PbS core/MAP shell QDs using the same methods mentioned earlier. In the viewpoint of charge transfer, we can expect the generated holes transfer without trap from PbS core to P3HT hole conductor through the MAP shell. The TRPL decay curves measured at the PL peak wavelength (1020 or 1170 nm) are shown in Fig. 5a. Next, we fitted the TRPL decay curves using a tri-exponential decay function. The fast component (τ1) in the tri-exponential decay function mainly attributed to the charge extraction to the transport layers. The rather slower components (τ2 and τ3) mainly due to the trap-assisted recombination or Auger recombination [35,36]. Fitting parameters obtained here are summarized in Table 2. Then, the electron transport efficiency for each sample was calculated using a method reported in the previous report [37] with the fitting parameters; the calculated results are shown in Fig. 5b. Since electron transport to mp-TiO_2_ competes with carrier recombination at surface defects, some of electrons cannot reach mp-TiO_2_. Therefore, the electron transport efficiency calculated here represents a ratio of the number of electrons reaching mp-TiO_2_ to the total number of electrons generated by photoexcitation before recombination [34]. The TRPL decays became quicker and the electron transport efficiency increased when the MAP shell thicknesses were increased from 0 to 0.32 nm. This points to improved electron transport to mp-TiO_2_ through MAP, probably because of passivated surface defects by introducing MAP shell layers for PbS QDs. However, increasing the MAP shell thicknesses to 0.45 nm resulted in an increase of PL lifetime and decrease of electron transport efficiency. This is because of too large shell thickness making carrier transport though MAP inefficient [38].

**Table 2:** Parameters obtained by fitting TRPL decay curves shown in Fig. 5a with a tri-exponential decay function [$f\left(x\right)=\sum_{i}A_{i}e^{-t/\tau_{i}}$]. The average lifetimes (*τ*_avg_) were calculated using an equation: $\tau_{\text{avg}}=\frac{\sum_{i}Ai∙\tau i}{\sum_{i}Ai}$. The electron transport efficiencies (*η*) calculated based on a method reported in Ref. [37] are also shown in this table.

MAP thickness (nm)	*A*_1_	*τ*_1_	*A*_2_	*τ*_2_	*A*_3_	*τ*_3_	*τ*_avg_	*η*_*inj*_ (%)
0	90.2	1.73	7.4	11.5	2.40	75.5	4.22	81
0.15	96.3	1.38	3.0	9.2	0.71	67.3	2.08	83
0.24	95.8	1.50	3.4	10.2	0.83	70.4	2.37	88
0.34	98.0	1.34	1.7	10.3	0.30	70.1	1.70	90
0.45	97.6	1.49	2.0	10.3	0.40	71.6	1.95	55

To gain further insight into carrier dynamics, we used a TPV measurement method. TPV techniques allow for direct monitoring of the bulk lifetime of photogenerated charge carriers, which defines the photovoltage of the solar cell. Consequently, TPV can be utilized to monitor charge carrier kinetics by identifying the charge carrier lifetime and determining the charge carrier density of the states in the device under relevant conditions. The result can reflect the trapping of the photo-generated charge and the quality of contact between different layers. PbS QD-sensitized solar cells with MAP shell layers revealed the longer recombination lifetime (τ_n_) at every *V*_OC_ compared with that of other solar cells (Fig. 5c). In addition, we provide a detailed comprehensive voltage variation data that is based on the measurement time, and is included in Figure S3of the supplementary material. This provides evidence that hole and electron transport to neighboring mp-TiO_2_ and P3HT layers is efficient and recombination of electrons and holes is slower in solar cells with MAP shell layers. These results agree well with the TRPL results.

From the above mentioned TRPL and TPV results, we can conclude that use of MAP shell layers passivates surface defects of PbS QDs, causing carrier recombination to reduce and hole and electron transport to mp-TiO_2_ and P3HT to increase. Therefore, the *J*_SC_ and FF values of solar cells were increased by introducing the MAP shell layers for the PbS QDs. In addition to the *J*_SC_ and FF, the *V*_OC_ values were increased as well, which is common in reported core/shell QD-sensitized solar cells. Yang et al. demonstrated that splitting of quasi-Fermi levels caused by reducing surface defects, leading to a low electron trap results in an increase of *V*_OC_ for core/shell QD-sensitized solar cells [20]. Speirs et al. introduced CdS shell layers for PbS QDs and found an increase of *V*_OC_ because of a band offset [19]. These Fermi splitting and band offset could be the possible sources of the increased *V*_OC_ in our devices. However, we need further studies to clarify more detailed factors affecting *V*_OC_.

**Fig. 6:**
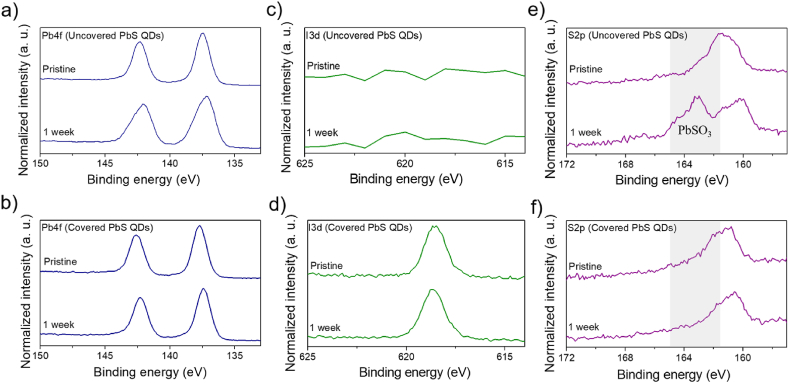
XPS profiles of as-prepared and stored PbS QDs without (a, c, and e) and with (b, d, and f) MAP shell layers of 0.34 nm. These XPS profiles include Pb_4f_ (a, b), I_3d_ (c, d), and S_2p_) (e, f) peaks. The storage condition was one week in air, with a temperature of 25–30 °C (room temperature) and a relative humidity of about 50–60%RH.

PbS QDs covered with MAP shells are expected to have better air stability than uncovered PbS QDs have. Therefore, we evaluated air stability of PbS QD samples in the absence and presence of MAP shell layers by XPS. We carried out XPS on the as-prepared QD samples. After storing these QD samples in air for one week, we measured their XPS profiles again. As can be seen in Fig. 6a and b, the MAP-covered and uncovered PbS QD samples exhibited the similar two Pb_4f_ peaks around 142.5 (4f_5/2_) and 137.5 (4f_7/2_) eV before and after the storage in air, which originate from Pb^2+^ ions of PbS or MAP. The uncovered PbS QDs, which were synthesized from PbI_2_ and Na_2_S, had no I_3d_ peak (Fig. 6c), indicating the effective removal of I^−^ ions from the samples during our S-SILAR method. On the other hand, the covered PbS QDs had a clear I_3d_ peak at about 619 eV (Fig. 6d), indicating the presence of MAP containing I^−^ ions. The I_3d_ peak with the similar intensity and position still existed even after the storage. This may mean that the MAP structure does not degrade significantly during the storage. Both the covered and uncovered PbS QDs had a S_2p_ peak before the storage, which are located at 160−162 eV and can be assigned to S^2−^ ions of PbS. After the uncovered PbS QDs were stored in air for one week, a peak originating from PbSO_3_ appeared at about 163 eV (Fig. 6e) while the XPS data was unchanged for the covered PbS QDs (Fig. 6f). These results provide clear evidence that the MAP shell layers can prevent oxidation from occurring in PbS QDs. In fact, our solar cells with the MAP shell layers demonstrated better air stability in comparison with those without the MAP layers (Figure S4).

## 4 Conclusions

**Fig. 7:**
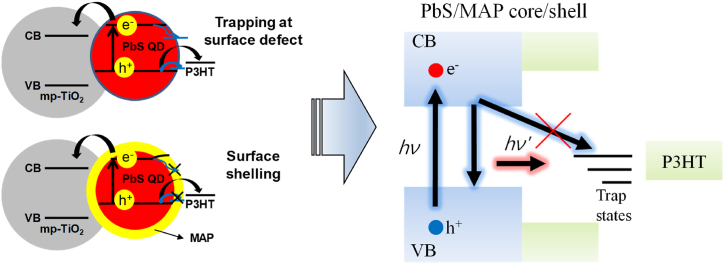
Schematic illustration showing carrier trapping at surface defects of a PbS QD and passivating surface defects by a MAPI_3_ shell layer. CB and VB stand for conduction and valence bands, respectively.

Based on the experimental observations, we propose that there are a number of defect states on surfaces of PbS QDs, which cause carrier recombination to occur and carrier extraction from devices (carrier transport to neighboring layers) to reduce as illustrated in Fig. 7. Therefore, it is important to passivate these defect states by use of the shell layers. In this study, we used MAP as the shell layers for PbS QDs and optimized MAP shell thickness. We demonstrated that use of the MAP shell thickness around 0.34 nm effectively passivates the surface defects along with enhanced carrier transport as proved with detailed analyses by TRPL, TPV, and XPS and, therefore, increased PCE from 0.7 to 4.1%. Additionally, we obtained improved air stability of solar cells by utilizing the MAP shell layers. These results will be valuable for establishing the basic physics of carrier dynamics in core/shell QD systems and fabrication of QD-sensitized solar cells with high efficiency and air stability.

## Author contribution statement

Conceived and designed the experiments: Gabseok Seo and Min-cheol Kim<a name = "Line_euclid5513277897_39">

Performed the experiments: Gabseok Seo and Shinhyun Kim.

Analyzed and interpreted the data: Gabseok Seo, Shinhyun Kim, Hyunseok Choi and Min-cheol Kim<a name = "Line_euclid5513277897_41">

Contributed reagents, materials, analysis tools or data: Min-cheol Kim.

Wrote the paper: Gabseok Seo and Min-cheol Kim.

## Data availability statement

Data will be made available on request.

## Declaration of competing interest

The authors declare that they have no known competing financial interests or personal relationships that could have appeared to influence the work reported in this paper.
