# Supplementary material for: Enhanced performance of lead sulfide quantum dot-sensitized solar cells by controlling the thickness of metal halide perovskite shells
Source: Heliyon. 2023 Sep 19;9(10):e20276. doi: 10.1016/j.heliyon.2023.e20276 (PMC10520828; doi:10.1016/j.heliyon.2023.e20276)
Supplement: Multimedia component 1 [file mmc1.docx]

**Supplementary material**

Enhanced performance of lead sulfide quantum dot-sensitized solar cells by controlling the thickness of metal halide perovskite shells

Gabseok Seo^a†*^, Shinhyun Kim^b†^, Hyunseok Choi^b^ and Min-cheol Kim^b*^

*^a^*Frontier Energy Solution Corporation, Seoul National University, Seoul 08826, Republic of Korea

*^b^*School of Mechanical Engineering, Pusan National University, Busan 46241, South Korea

^†^These authors contributed equally to this work

***corresponding author: [photoacoustic.image@gmail.com](mailto:photoacoustic.image@gmail.com), [mckim90@pusan.ac.kr](mailto:mckim90@pusan.ac.kr)


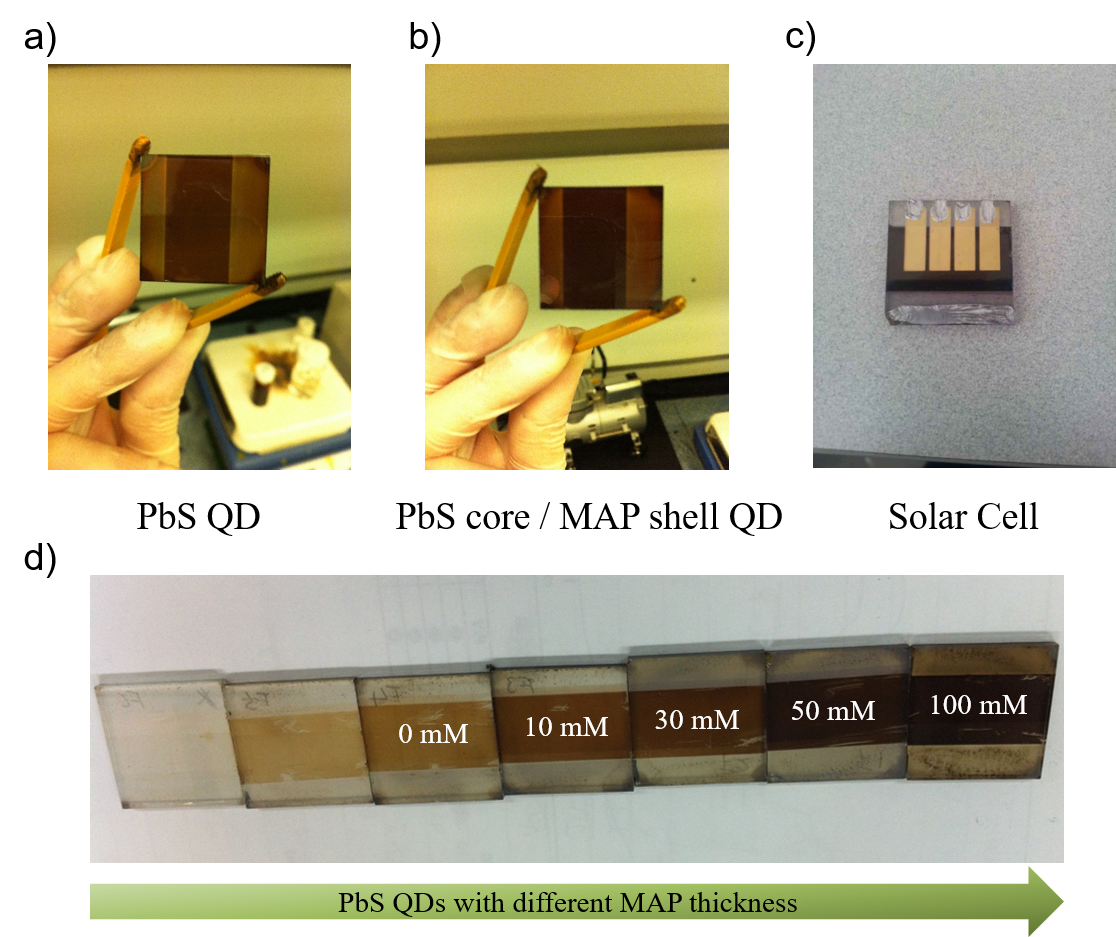


Figure S1. Pictures of mp-TiO_2_ films with (a) PbS QDs and (b) PbS core/MAP shell QDs. (c) represents a solar cell with PbS core/MAP shell QDs. As can be seen in (d) sample color became darker when MAP shell thicknesses were increased.

|  |
| --- |
|  |


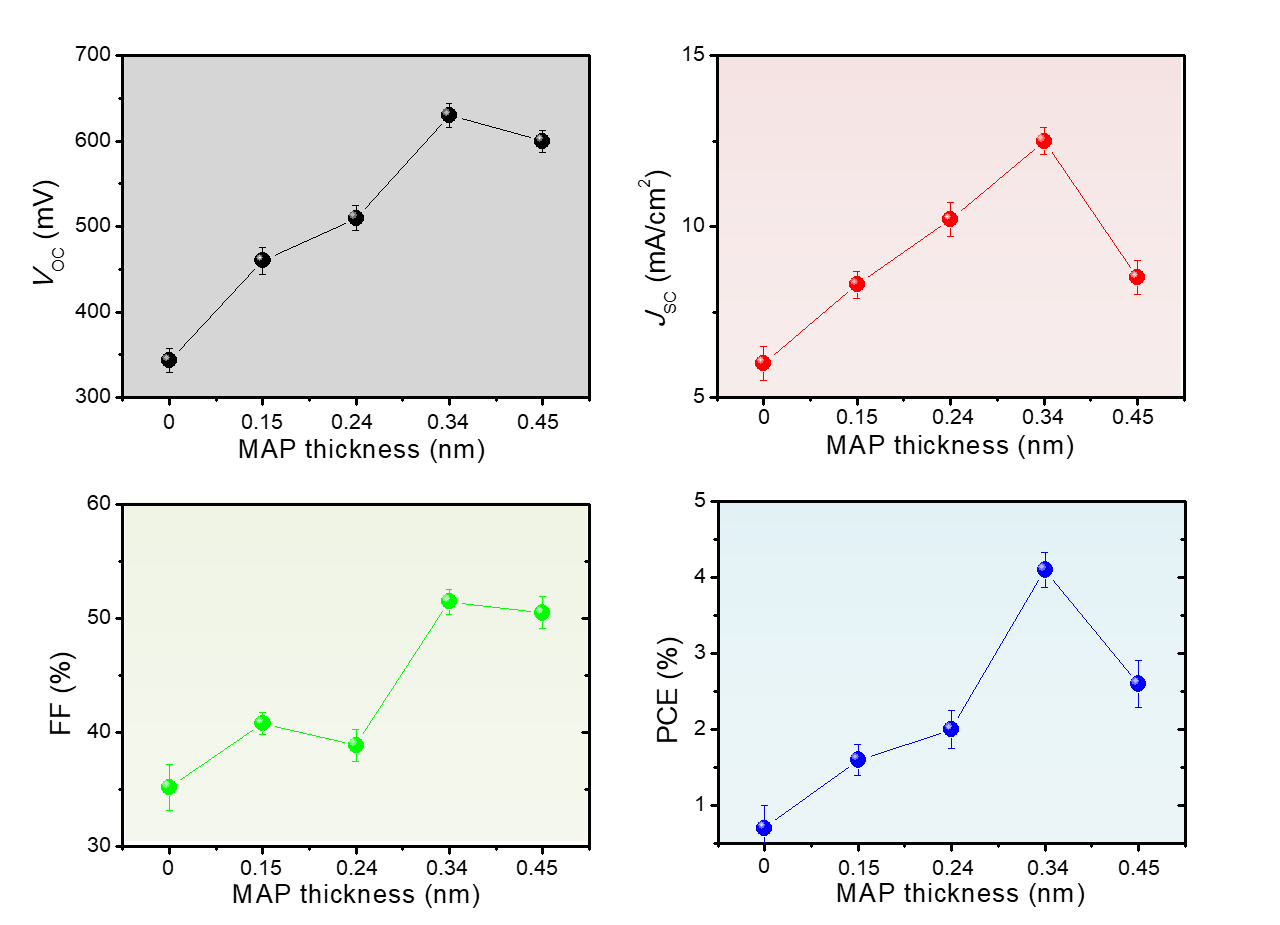


Figure S2. Histograms representing 50 samples of *V*_OC_, *J*_SC_, FF and PCE values as a function of MAP shell thickness for PbS QD-sensitized solar cells.


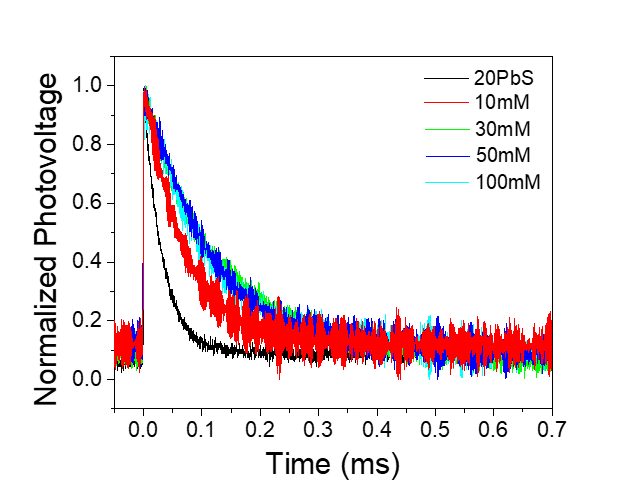


Figure S3. The transient photovoltage (TPC) decay measurement data as a function of open-circuit voltage (V_OC_) of the 20PbS and 20PbS with MAP shell (10~100mM).


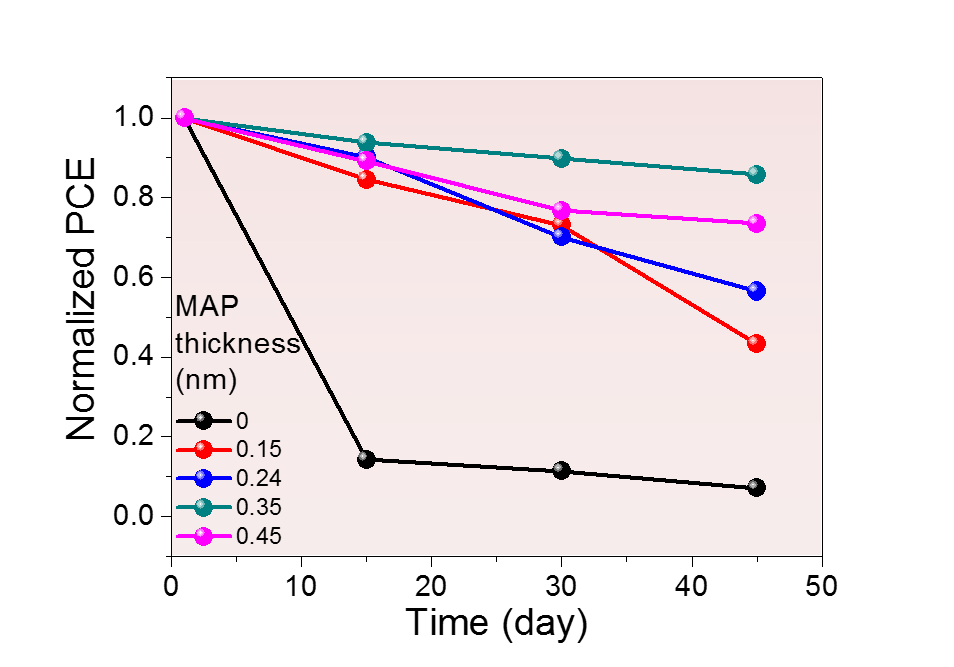


Figure S4. Evolution of PCE for solar cells in air without light illumination. The storage condition was one week in air with a temperature of 25−30 ºC and a relative humidity of about 70−80 %RH.
